# A Corneal Biomechanical Study Measured with a Scheimpflug Dynamic Analyser in Soft Contact Lens Wearers

**DOI:** 10.3390/life13122313

**Published:** 2023-12-08

**Authors:** Alfredo López-Muñoz, Isabel López-Castaño, Úrsula Torres-Parejo, Marta-C. García-Romera

**Affiliations:** 1 Department of Physics of Condensed Matter, Optics Area, Vision Sciences Research Group (CIVIUS), Pharmacy School, University of Seville, 41009 Seville, Spain; isabel270600@gmail.com (I.L.-C.); mgarcia118@us.es (M.-C.G.-R.); 2 Research & Development Department (Miranza Virgen de Luján®), Ophthalmology Center, 41011 Seville, Spain; 3 Department of Statistics and Operations Research, University of Grenade, 18071 Grenade, Spain; ursula@ugr.es

**Keywords:** myopia, soft contact lenses, corneal biomechanics, Scheimpflug technology, Corvis ST^®^

## Abstract

The aim of this study was to evaluate the biomechanical changes in the cornea after wearing soft contact lenses (CLs) in healthy myopic patients measured with a Corvis ST^®^ (CST, Oculus Optikgeräte GmbH, Wetzlar, Germany) analyser. This prospective, cross-sectional, single-centre study was performed on twenty-two Caucasian patients aged between 19 and 24 years (20.64 ± 1.21 years) range. Five device-specific biomechanical parameters, the central corneal thickness (CCT), and biomechanically corrected intraocular pressure (bIOP) were measured prior to fitting and one month after CL wear. Differences between the means of the deflection amplitude ratio (DA Ratio) and the standard deviation of the DA Ratio (SD DA Ratio) pre- and post-CL wear were found to be significant (*p* value = 0.002 in both cases). Significant differences were found between pre- and post-CL wear values in CCT (*p* value = 0.013). For all other biomechanical measures, no significant differences were observed before and after treatment. A significant association was found between changes in bIOP and classification according to changes in Int. Radius (*p* value = 0.047) and SSI (*p* value = 0.026) standard deviations. The corneal biomechanical indices provided by CST demonstrate that the fitting of soft CLs is a safe optical compensation method for the stability of corneal stiffness. No significant differences were found pre- and post-CL wear in the assessment of bIOP.

## 1. Introduction

The shape of the cornea is a determining factor in ocular refraction but is itself determined by its biomechanical properties. The cornea must be soft enough to expand into the spherical hemisphere but rigid enough to hold its shape and resist intraocular pressure (IOP) [1]. Biological properties, such as healing responses and biomechanics, are essential in determining and maintaining corneal transparency, as well as geometric and optical properties [2].

Most biomechanical studies to date have focused on the stroma, which constitutes 90% of the total thickness of the cornea and is generally considered to be the primary supporting layer of the cornea. Studies have demonstrated the complex nature of the stroma and the regular diameter and spacing of the collagen fibrils, as well as their influence on corneal transparency and biomechanical behaviour [3].

Notably, the cornea maintains a delicate and complex balance between stiffness, strength, elasticity, and overall strength to withstand internal and external forces that constantly compress it, distort its shape, or threaten its integrity [4]. This is measured by corneal biomechanics, which has emerged as a research and development topic in modern ophthalmology due to its many potential applications [5]. 

Biomechanics is commonly defined as the application of mechanics to biology. However, the term is better described as an extension and development of mechanics with the goal of understanding physiology and physiopathology, as well as the diagnosis and treatment of injuries and diseases due to the intricate and diversified behaviour of biological structures and materials [6].

Corneal biomechanics is the study of the structure of the cornea by defining the physical and mathematical principles that can predict the dynamic response of the cornea to physiological and/or pathological conditions through behavioural patterns or model definitions of the corneal tissue. Corneal biomechanics is the science that deals with the balance and deformation of tissue subjected to any force. It studies the function and structure of the cornea and forms a basis for predicting its dynamic response in physiological and pathological conditions [7].

The capability to measure the biomechanical properties of the cornea in vivo is of great clinical importance as it helps to improve many treatment and management procedures that mechanically interact with or affect the eye. Examples include measuring IOP for effective glaucoma management [8]; planning refractive surgery [9]; determining keratoconus risk [10]; and optimising various protocols for collagen cross-linking treatments or evaluation [11], including preoperative evaluations for the retreatment of refractive surgery. The mechanical interaction between the lens and the anterior segment is not currently considered in the selection of intracorneal ring implants or even in the design of soft contact lenses [12]. Additionally, corneal biomechanical analysis has been suggested as a potentially relevant factor in orthokeratology (OK), but the role of corneal biomechanical properties in predicting the correction obtained with this refractive compensation option is unclear [6].

Interest in the use of biomechanical principles in the cornea has increased significantly in recent years with the aim of better understanding corneal behaviour and improving the safety and efficacy of various ocular treatments or refractive techniques [13].

An in vivo evaluation of corneal parameters is essential to understand corneal behaviour under physical stress. However, in clinical practice, it is not easy to accurately evaluate the behaviour of the cornea under stress and use the results to estimate some mechanical properties of the cornea [13]. However, until recently, the evaluation of the biomechanical properties of the cornea was limited to ex vivo laboratory studies and mathematical models of the cornea [14].

There are still a limited number of techniques developed and tested to characterise corneal biomechanics with potential application in clinical practice. Two instruments are currently available to characterise the biomechanical properties of the cornea in clinical settings, namely the Ocular Response Analyser (ORA; Reichert Inc., Depew, NY, USA) and the Corvis Scheimpflug Technology (CST, Oculus Optikgeräte GmbH, Wetzlar, Germany), based on the measurement of corneal deformation using the Scheimpflug technique. Both have unique parameters that describe corneal biomechanics, but their relationship to standard mechanical properties is unknown and is not associated with a specific biomechanical model. Therefore, there is inconsistency in the definition of some fundamental biomechanical parameters, such as viscosity or elasticity, to characterise the biomechanical properties of the cornea. As a result, comparative analysis between studies using different technologies is difficult [15].

The ORA was presented as the first equipment to assess the biomechanical behaviour of the cornea in vivo at the 2005 ESCRS meeting (Lisbon, Portugal) [16]. The ORA is a modified non-contact tonometer (NCT) initially designed to provide more accurate IOP measurements through corneal biomechanical compensation. It analyses the behaviour of the cornea during bidirectional applanation induced by an air jet and generates estimates of corneal hysteresis (CH) and the corneal resistance factor (CRF) along with a set of 36 waveform-derived parameters [14]. The ORA combines an air puff with an infrared transmitter and receiver. This device can evaluate corneal deformation only indirectly based on infrared signals [17].

The photoelectric coherence detection system monitors the curvature of the cornea with a central diameter of 3.0 mm during a 20 ms measurement [18]. In this system, the maximum air pressure generated varies from the first stabilisation event. The maximum pressure of the ORA is adjusted from test to test so that eyes with early initial loading and typically low IOP receive a lower maximum pressure, and eyes with high IOP receive a larger maximum pressure bladder [6]. The measurement involves automatic alignment with the top of the cornea and triggers the air puff. The measurement takes approximately 25 ms. The cornea deforms due to air pressure (internal phase), and the first flattening occurs when the pressure is recorded (P1). The cornea takes a concave shape, until the air pressure decreases, allowing the cornea to gradually return to its normal shape. During the exit phase, it undergoes a second delamination state, where the pressure (P2) is recorded again. Both abrasion events are recognised by peaks in the corneal reflex signal corresponding to two independent pressure values in the air puff pressure profile. These pressure measurements (P1 and P2) form the basis of the first-generation variants reported from the original ORA program [16].

The average of the two applanation pressures was correlated with Goldmann tonometry results in an internal study with the aim of providing (linear) calibration coefficients for reporting intraocular pressure and CH in millimetres of mercury (mmHg). The procedure has been described [18].

The CST, the analytical tool used in this study, was later introduced as NCT. This device uses an ultrahigh-speed (UHS) Scheimpflug camera to monitor the corneal response to air pressure pulses and uses the acquired image sequences to estimate IOP and strain response parameters [14].

The CST has been commercially available since 2011 and is based on UHS dynamic Scheimpflug imaging technology. It measures IOP, central corneal thickness (CCT), and corneal biomechanical parameters by directly observing and imaging corneal deformation in response to a standard puff of air in real time [19]. The instrument is ergonomically designed with an adjustable head control and chin rest. The patient is positioned comfortably with the chin and forehead positioned appropriately. The patient is asked to focus on the central red LED (light-emitting diode). A front camera with a keratometer-type projection system is installed to focus and align the corneal apex. The test is programmed to trigger automatically when synchronisation with the first corneal Purkinje reflex is achieved. Manual triggering is also possible [2].

The UHS Scheimpflug camera uses more than 4300 frames per second to monitor the corneal response to a collimated puff of air measured in a fixed profile with a symmetrical configuration and a fixed maximum internal pump pressure of 25 kPa. UHS Scheimpflug cameras are equipped with blue light LEDs (455 nm, no UV) and cover 8.5 mm horizontally with a slit. The exposure time is 30 ms, and 140 digital images can be acquired. Each image has 576 pixels [2].

In addition to IOP, corrected IOP (corrected IOP based on the Dresden correction table), and CCT, the following parameters are measured: time to the first (A1) and second (A2) lamination (time to reach the first and second lamination, respectively); A1 and A2 lengths (the length of the segment flatness in the Scheimpflug image during the first and second flattening); A1 and A2 velocities (the velocity of corneal movement in inner and outer flattening); and features of the highest concavity, including time to reach maximum stress (hours), strain amplitude (DA), and distance between the peak point of the curvature (PD) and the radius of the curvature [19].

The development of the new biomechanical principles of ocular structure is an emerging area of research in optometry and ophthalmology. This is a challenge that must be met in order to create more appropriate in vivo biomechanical models of the cornea and to define appropriate predictive models of corneal behaviour. These tools will allow the clinician to predict the clinical outcomes of various ocular treatments before they are performed, thus allowing for their optimisation [20].

On the other hand, the adaptation of contact lenses (CLs) on the ocular surface causes a multitude of physical corneal changes, modifying its curvature and tear quality. From the first hours of wear, corneal alterations are recorded that lead to the appearance of allergic, infectious, anatomical, and metabolic phenomena resulting in discomfort or discomfort for the wearer, with infection being the most serious complication [21]. However, there are limited data on corneal biomechanical changes after the daily wear of soft CLs. There are several reports of changes in corneal topography [22], changes in anterior corneal topography [23], and central corneal oedema [24] after CL use.

A possible mechanism for changes in biomechanical properties has been attributed to corneal stromal oedema after CL use, which increases the distance between collagen fibrils and affects the biomechanical function of the cornea [25]. Furthermore, another possible hypothesis regarding the repolarisation of corneal tissue and the resulting changes in corneal biomechanical behaviour may be related to local alterations in inflammatory cytokines and chemokines after CL use [26].

The scientific literature contains several studies on the characterisation of corneal biomechanics in different study designs, with investigations using both the ORA and the CST [6]. Regarding corneal biomechanics related to the wearing of soft CLs, we found studies utilising the ORA, but to our knowledge, there are limited reports about the relationship between the use of soft CLs and corneal biomechanics using the CST [27].

Therefore, we consider it of utmost importance to understand the structural and biomechanical changes in the cornea after wearing CLs in our research of hydrophilic material because this may have important clinical implications, especially for patients whose properties are already altered before use, as in the case of pathological corneas, as well as for the development of new CLs with different uses or applications. Thus, the need for this project arises.

## 2. Materials and Methods

### 2.1. Design

This prospective, cross-sectional, single-centre study was conducted between February 2023 and May 2023 at the facilities of the Faculty of Pharmacy (Department of Optics and Optometry) of the University of Seville, Spain. The study complied with the standards established by the Declaration of Helsinki and the Andalusian Ethics Committee Council. After explaining the nature of the study, informed consent was obtained from each subject.

### 2.2. Subjects

Twenty-two Caucasian patients belonging to the student community of the University of Seville were recruited. To avoid any bias, only one eye from each participant was randomly selected for inclusion in this study [28]. Inclusion criteria were as follows: (1) 18 years of age; (2) habitual CL wearer; (3) simple or compound myopic refractive error (with astigmatism); (4) cessation of CL wear for at least 7–21 days among hydrophilic and rigid gas permeable lens wearers, respectively, with special topography follow-up in case of corneal moulding until disappearance or topographic stabilisation; and (5) the acceptance of participation in the study and ability to understand the informed consent and the subsequent signature of the consent form. Exclusion criteria were as follows: (1) collagen or autoimmune diseases; (2) being pregnant; (3) previous intraocular or corneal surgery; (4) ocular pathologies; (5) corneal dystrophies and degenerations; (6) dry eye syndrome; (7) persistent epithelial defects; (8) history of herpetic corneal ulcer; (9) central corneal leukoma; (10) topographic map compatible with subclinical keratoconus or other corneal ectatic disorder; and (11) current antiglaucomatous or hypotonic treatment.

### 2.3. Contact Lenses

The lens used by the subjects were Lens 55^®^ UV (Ocufilcon D4 (19) (55%); Servilens (Granada, Spain), https://www.lens55.com/documents/catalogo_Servilens_V20.pdf, accessed on 1 January 2023), a hydrogel lens with a thin rim design, performing well in terms of comfort and parameters.

CL prescriptions were determined based on the refraction of the spectacle distance and modified, if necessary, by taking into account the vertex distance. The lenses were fitted binocularly, and the same type of lens was used in each eye. Each pair of lenses was worn for 10 min to settle on the eye before starting the measurements and then worn for approximately half an hour during the assessment of distance visual acuity. After the settling period, the CL fit was checked and confirmed to be acceptable (<1.0 mm of decentration, <1.0 mm of movement) before proceeding with the assessment [29]. Thus, the wearers’ initial response to the CLs was assessed.

### 2.4. Procedure

Once the informed consent form had been signed, each study subject underwent a pre-visit in which, in addition to collecting the relevant data from the clinical history, they underwent a complete ophthalmological examination with the following diagnostic tests: optically corrected visual acuity (BCVA) according to the Snellen decimal scale; subjective and objective refraction without cycloplegia; corneal topography and axial length measurement with Pentacam AXL^®^, a device with a single rotation Scheimpflug camera with version 6.08r19 software produced by Oculus Optikgeräte in Wetzlar, Germany; corneal biomechanical analysis using Scheimpflug technology via the CST analyser (Oculus Optikgeräte GmbH, Wetzlar, Germany); and a slit-lamp anterior pole examination. Another visit was scheduled for CL fitting and a final examination one month after CL wear, at which time the biomechanical parameters were remeasured. 

All measurements with the CST were performed by the same technicians and recorded with automatic release to ensure the absence of examiner dependence. If the examination quality (QS) box showed any type of alteration, this was identified in the device software; to be considered an optimal image, the quality factor had to be higher than 95% (this figure may be lower due to the presence of artifacts in the image, eye blinking, or insufficient eye opening by the patient); otherwise, the necessary correction was made, and the acquisition was repeated. Only CST exams with an “OK” quality rating were included in the analysis, excluding alignment errors. Flicker errors were also excluded. In all cases, 3 measurements were taken per patient, and the mean was calculated.

**Figure 1:**
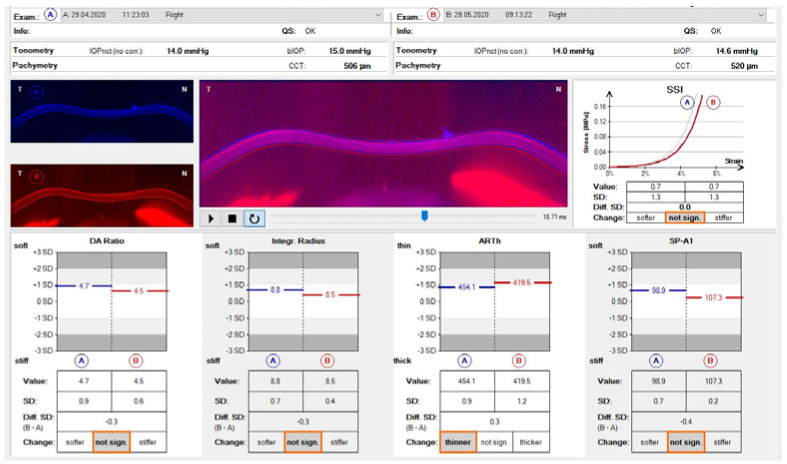
Comparison pre- and post-contact lens wear of biomechanical Corvis ST^®^ parameters. DA Ratio = deflection amplitude ratio; Integr. Radius = integrated radius; ARTh = Ambrósio horizontal relational thickness; SP-A1 = stiffness parameter-A1; SSI = stress–strain index.

The variables included in this study are the most reproducible parameters of the CST, as collected in the Biomechanical Comparison Display (Figure 1): (1) the deflection amplitude ratio (DA Ratio): This is the ratio between the central corneal deflection and the average of two points located at 1.0 mm (DA Ratio 1) or 2.0 mm (DA Ratio 2) either side of the centre. Stiffer corneas would have a lower DA Ratio because the centre of the cornea and the cornea at 1.0 or 2.0 mm deflect at the same time, whereas a higher DA Ratio indicates that the central cornea deflects more than the average of the other two points, corresponding to softer tissue; (2) the Ambrósio horizontal relational thickness (ARTh): Corneal thickness is measured using the horizontal Scheimpflug image. This allows the rate of increase in corneal thickness from the apex to the nasal and temporal sides to be calculated. The characterisation of the thickness profile allows for the calculation of the Ambrósio relational thickness across the horizontal meridian, which is a relative simplification of the tomographic relational thickness calculations also provided by the Pentacam; (3) the stiffness parameter-A1 (SP-A1): This is defined as the pressure at first flattening, which is the difference between the air bubble pressure at the corneal surface and the bIOP, divided by the DA Ratio. It is determined from the displacement of the apex from rest to the first flattening. This value has been clinically proven to be useful in assessing KC with the highest sensitivity and specificity of any of the parameter values. Higher values indicate stiffer corneas; (4) integrated radius (Int. Radius): This is a dynamic corneal deformation response parameter representing the reciprocal of the radius at the state of maximum corneal concavity. A larger concave radius is associated with greater resistance to deformation, i.e., a stiffer cornea. The larger the integrated inverse radius and the maximum inverse radius, the lower the resistance to deformation and the lower the corneal stiffness; and (5) the stress–strain index (SSI): This is a new parameter for estimating the material stiffness of corneal tissue that is independent of IOP and corneal geometry. The stress–strain curve describes the elastic properties of the cornea. The curves are shifted to the right if the cornea is soft and to the left if it is stiff. The SSI index describes the position of the curve. A value of 1 indicates average elasticity, a value less than 1 indicates softer behaviour, and a value greater than 1 indicates stiffer than average behaviour. The secondary variables were (1) the central corneal thickness (CCT) and (2) the biomechanically corrected intraocular pressure (bIOP). None of the variables have units, and moreover, they do not have standardised values. However, the standard deviation (SD) PRE–POST was compared, and depending on the variable, the change was qualified as not significant, softer/stiffer, or thinner/thicker (Table 1). All measurements with the CST were taken by the same technicians and captured via automatic release to ensure no examiner dependence. To be considered an optimal image, the quality factor had to be higher than 95%. Other parameters that were measured, not included as study variables, were the spherical equivalent (SE), with a mean value of −3.63 D ± 0.41 D (−9.25 D, −0.75 D); the axial length of the eyeball (AXL), with a mean value of 25.16 mm ± 0.17 mm (24.82 mm, 25.50 mm); and the mean keratometry (Km), calculated as the average of K1 and K2 within the 3 mm central optical zone, with a mean result of 7.80 mm ± 0.49 mm (7.28 mm, 8.25 mm). In all cases, 3 measurements were obtained per patient, and the mean value was used.

### 2.5. Data Analysis

The collected data were reviewed for consistency and correctness. Subsequently, statistical analysis was carried out using SPSS software for Windows version 22.0 (IBM, SPSS, Inc., Chicago, IL, USA). The normality of both the variables that record the biomechanical values and those that record the standard deviations of these values was checked using the Shapiro–Wilk test. For the variables that followed a normal distribution, the *t*-test for related samples was employed. For those variables for which it was not possible to assume normality, non-parametric techniques were used, specifically the Wilcoxon test. For all statistical tests, a *p* value < 0.05 was considered statistically significant. The statistical analysis of the collected data under the guidelines of the clinical protocol allowed us to draw and establish the conclusions of the study, thus quantifying the biomechanical impact on the use of soft CLs.

## 3. Results

In total, 22 eyes (6 right (27.3%) and 16 left (72.7%)) of 22 patients (mean age, 20.64 ± 1.21 years, range 19–24 years) were included. There were 7 males (31.8%) and 15 females (68.2%). Descriptive data for biomechanical measurements pre- and post-CL wear are presented in Table 2.

**Figure 2:**
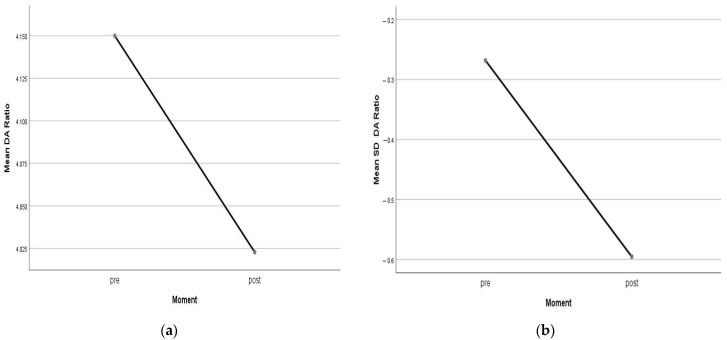
(**a**) Differences between the mean of the DA Ratio plot pre- and post-contact lens wear; (**b**) Differences between the mean of the SD_DA Ratio plot pre- and post-contact lens wear.

**Figure 3:**
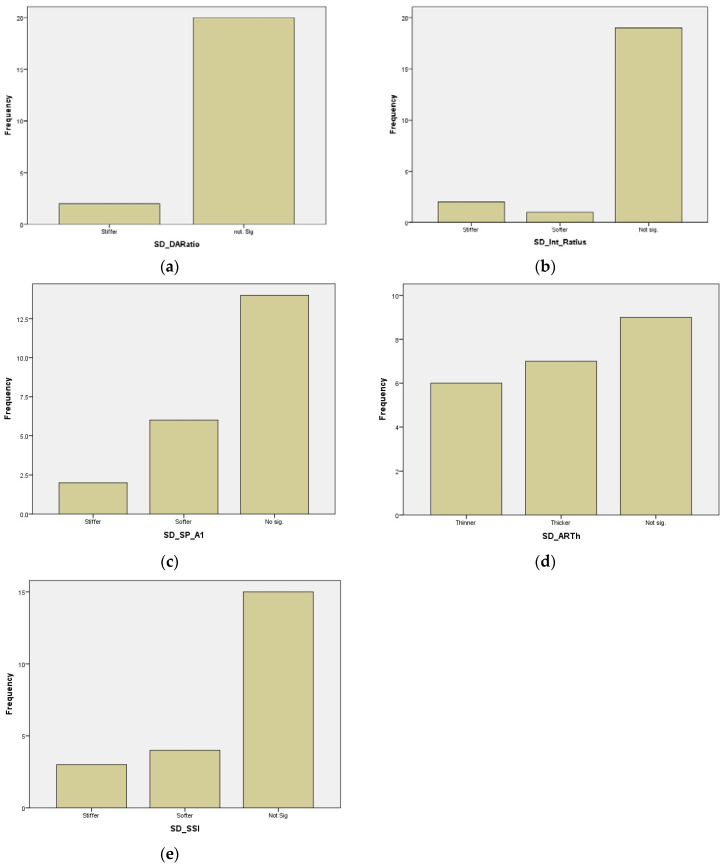
Histograms of classification according to changes in different biomechanical measures pre- and post-contact lens wear: (**a**) SD_DA Ratio; (**b**) SD_Int_Ratius; (**c**) SD_SP_A1; (**d**) SD_ARTh; (**e**) SD_SSI.

Differences between the means of the DA Ratio and the SD DA Ratio pre- and post-CL wear (Figure 2) were found to be significant according to Student’s *t*-test for the related samples (*p* value = 0.002 in both cases). Similarly, significant differences were found between pre- and post-CL wear values in CCT according to the Wilcoxon test (*p* value = 0.013), indicating corneal thinning. For all other biomechanical measures, no significant differences were observed before and after treatment. Table 3 presents the summary of the changes, showing the 95% confidence intervals for the mean SD difference (pre–post) at normality assumptions and the classification value for these changes, according to which the patients are classified as shown in histograms in Figure 3. The biomechanical changes correlating significantly through Pearson’s r correlation coefficient were changes in the DA Ratio and ARTh (r = 0.442, *p* = 0.039), changes in the Int. Radius and SP-A1 (r = 0.475, *p* = 0.025), and changes in SP-A1 and SSI (r = 0.434, *p* = 0.044), showing direct linear associations. Similarly, an inverse linear correlation was found between the spherical equivalent (SE) and axial length (AXL) (r = −0.633, *p* = 0.002). No significant associations were found for any variable according to age, sex, or eye (right or left). A significant association was found according to the chi-square test between changes in bIOP and classification according to changes in the Int. Radius (*p* value = 0.047) and SSI (*p* value = 0.026) standard deviations.

## 4. Discussion

Most studies analysing changes in biomechanical properties, especially after different corneal refractive procedures, have been performed with the ORA, as it was the first to be available. Its clinical introduction was extremely important because it was the first time that the biomechanical response of the cornea to a perturbation could be measured in vivo using a puff of air to deform it. However, basic misconceptions have been perpetuated, obscuring the interpretation of the results, including the desire to biomechanically characterise the cornea with a single number that may answer clinical questions about corneal stiffness or basic corneal weakness [20].

Similarly, when the CST became available, there was a new wave of studies, from the first in 2014, in which Hassan et al. compared the results of PRK and LASIK techniques [30], until 2017, when the same team discussed the effects of FEMTOLASIK and PRK [31].

Interest in corneal biomechanics was spreading as the instrument evolved, providing new parameters. Thus, authors such as Yang et al. decided to compare these new parameters in healthy eyes undergoing LASIK surgery, patients with post-LASIK ectasia, and patients with keratoconus [32].

Regarding CL wear, most studies are related to OK and carried out with the ORA. This is the case for Chen et al. [33], who determined an alteration in biomechanical properties such as a lower corneal resistance factor (CRF) as the duration of orthokeratology lens wear increased. On the other hand, Manuel González-Méijome et al. found a faster recovery effect in less resistant corneas, correlating corneal hysteresis (CH) with changes in keratometry and CCT during lens wear and reporting a need for further studies to determine these changes [34].

Other previous studies have investigated corneal biomechanical changes measured with the ORA after refractive lens correction. Corneal hysteresis (CH) and the corneal resistance factor (CRF) were shown to be reduced 6 months after OK lens wear, and corneal stiffness was positively associated with myopia reduction in terms of the spherical equivalent (SE). Reduced CH and CRF indicate a lower viscoelastic property of the cornea [35]. In the study by Lin et al., the changed parameters measured with the CST all indicated a more deformable cornea after Defocus Incorporated Soft Contact (DISC) lens wear than OK lens treatment [36]. Furthermore, in the study by Wan et al., baseline CH was negatively associated with AXL elongation after single vision spectacle (SVS) wear, and corneal biomechanics were believed to predict the rate of AXL elongation [37]. However, CH and CRF were not associated with AXL elongation in the OK group [37]. This may be because the ORA could provide less information about corneal biomechanical properties, and the CH and CRF measured with the ORA varied widely among individuals [38].

The Corvis ST^®^ measured more dynamic corneal biomechanical parameters with high repeatability [39]. The results of the study of Lin et al. showed that several baseline CST parameters associated with AXL elongation and the related parameters differed after DISC and OK lens treatments [37]. In this study, despite having measured AXL, inversely correlated with myopic SE (r = −0.633), and not having analysed the change pre- and post-CL wear, no association was found with biomechanical variables.

As corneal swelling, shape alterations, refractive stability, and induced optical aberrations are crucial factors for normal vision [1], there has been an increasing effort to understand how corneal biomechanics is affected by soft CL wear.

For soft CLs, most studies have also used the ORA. Cankaya et al. performed an analysis with the aim of comparing CH and CRF with and without wearing CLs [40], concluding that CH did not show a trend of change with the use of CLs. Conversely, Somayeh Radaie Moghadam et al. and Lau and Pye reported a decrease in CH one month after fitting and immediately after using CLs [41,42]. On the other hand, the CRF showed higher values in patients wearing CLs, with a statistically significant difference associated with corneal remodelling due to the chronic use of CLs. Somayeh Radaie Moghadam et al. (2016) and Lau and Pye (2011) found a decrease in CRF when CL use was discontinued. In our case, corneal weakening was observed after CL wear in a small percentage of wearers, although in the majority, there was no statistical significance.

Considering the studies performed with the CST, there is controversy in the literature with regard to changes in the CST after the use of soft CLs, as Braun and Penno indicated that this value decreased in relation to the control population [24], whereas authors such as Cemal Çavdarli and Peyman et al. observed a null impact [27,43]. By evaluating these long-term changes, Yeniad et al. showed thickening after one month of use and thinning after 6 months. In this study, an increase in corneal thickness (*p* value < 0.05) was observed in the measurement after one month of wear [44].

Similar to the study by Peyman et al. [27], in the present study, we also found no significant differences in corneal biomechanical parameters after one month of CL use. Changes in the DA Ratio and ARTh, Int. Radius and SP-A1, and SP-A1 and SSI correlated directly, with SE and AXL correlating inversely.

Sapkota et al. studied the effect of soft CL on IOP, observing a reduction in both Goldmann-correlated intraocular pressure (gIOP) and compensated intraocular pressure (cIOP), as measured with the ORA, which was approximately 1.02 mmHg during the first month. The authors concluded that these changes were significantly related to the lens type, i.e., daily or monthly disposable, but not to the wearing pattern [45]. In our study, the bIOP value decreased (*p* value > 0.05). 

Although in the present project, only significant differences were found in both the DA Ratio and its SD, with their mean values being higher before than after the wear of CLs, this difference did not exceed a value of 1; therefore, the change could not be classified as “stiffer”. Our results are in contrast to those of Marcellán et al., who found that the biomechanical parameters and physical properties of the cornea can be altered with the use of soft CLs, although in this case, it was the use of silicone hydrogel CLs (SiH-CLs), and the measurement was performed with the ORA [46].

As far as IOP is concerned, Lau and Pye reported that the biomechanical overestimation measured via applanation tonometry was due to temporal hydration or induced corneal oedema as a result of hydrogel soft CL wear [37]. This overestimation is due to an increase in corneal stiffness caused by corneal hyper-hydration. In the study by Marcellán et al., the longer period of follow-up (20 days) revealed a greater decrease in IOP and a statistical increase in CH, while no induced oedema or increase in corneal stiffness (CRF) was observed [46]. This inverse relationship may be explained by La Place’s law: as the IOP increases, stiffness is higher, and the viscous damping reduces, i.e., IOP and CH are negatively correlated [47].

If IOP decreases while the CCT remains stable, the viscoelastic response increases for mechanical compensation to maintain corneal resistance. This indicates that while the elastic property is weakening, the viscoelastic response compensates to maintain corneal integrity. The viscosity increase compensates for the corneal biomechanics and maintains ocular stability [41]. However, in this study, significant differences were found between pre- and post-CL wear values in the CCT, with a thinning of 11 µm.

In the present study, the fact that the bIOP values before and after wearing soft CLs were very similar, with a change of −0.53 mmHg, indicates that the use of this type of CLs does not alter the bIOP value and that the accuracy of the CST for IOP measurement in each patient avoids the need to adjust traditional tonometric measurements after refractive surgery or CL wear since IOP assessment is a critical factor in the diagnosis and management of glaucoma.

## 5. Conclusions

Uncovering the biomechanical properties of the human cornea is essential for comprehending the onset of corneal diseases and devising novel treatments. Our research has revealed that the fitting of hydrogel soft CLs is a safe optical compensation method for the stability of corneal stiffness in healthy patients with myopia, as demonstrated by the corneal biomechanical indices provided by the CST.

No significant differences were found pre- and post-CL wear in the assessment of bIOP. It is confirmed that the CST is an objective tool for IOP measurement since IOP assessment is a critical factor in the diagnosis and management of glaucoma. Our findings could have an impact on the management of glaucoma progression and ocular hypertension.

We believe that our study would benefit from a control group either not wearing CLs or a group with newer materials lenses such as silicone hydrogel. 

An advanced understanding of the biomechanical properties of the cornea would be needed for those patients with mild or subclinical forms of ectatic corneal disease who can still compensate for their refractive error by wearing soft CLs.

## Figures and Tables

**Table 1:** Classification of the change in standard deviation of biomechanical parameters post–pre (B-A) of the Biomechanical Comparison Display software version 1.6r2554.

	B-A Not Significant	B-A Stiffer	B-A Softer
DA Ratio	±1.0	>−1.0	>+1.0
Int. Radius	±0.7	>−0.7	>+0.7
SP-A1	±0.8	>−0.8	>+0.8
SSI	±0.4	>−0.4	>+0.4
	**B-A Not Significant**	**B-A Thicker**	**B-A Thinner**
ARTh	±0.3	>−0.3	>+0.3

DA Ratio = deflection amplitude ratio; Int. Radius = integrated radius; SP-A1 = stiffness parameter-A1; SSI = stress–strain index; ARTh = Ambrósio horizontal relational thickness.

**Table 2:** Descriptive analysis of biomechanical data, bIOP and CCT, pre- and post-contact lens wear.

	Variables				
	Mean ± SD			Mean ± SD	
DA Ratio	Pre	4.15 ± 0.32	SD_DA Ratio	Pre	−0.27 ± 0.78
	Post	4.02 ± 0.29		Post	−0.60 ± 0.68
Int. Radius	Pre	7.57 ± 1.05	SD_Int. Ratius	Pre	−0.43 ± 0.93
	Post	7.38 ± 0.83		Post	−0.59 ± 0.75
ARTh	Pre	521.14 ± 81.60	SD_ARTh	Pre	0.33 ± 0.66
	Post	528.77 ± 106.75		Post	0.29 ± 0.85
SP-A1	Pre	112.77 ± 15.50	SD_SP-A1	Pre	−0.04 ± 0.82
	Post	108.82 ± 12.69		Post	0.16 ± 0.69
SSI	Pre	0.97 ± 0.14	SD_SSI	Pre	0.20 ± 0.58
	Post	0.95 ± 0.14		Post	0.17 ± 0.66
bIOP	Pre	16.14 ± 2.67	CCT	Pre	556.00 ± 91.38
	Post	15.61 ± 1.85		Post	545.23 ± 21.75

DA Ratio = deflection amplitude ratio; Int. Radius = integrated radius; ARTh = Ambrósio horizontal relational thickness; SP-A1 = stiffness parameter-A1; SSI = stress–strain index; bIOP = biomechanically corrected intraocular pressure; CCT = central corneal thickness; SD = standard deviation.

**Table 3:** Summary of changes in corneal biomechanical parameters, bIOP and CCT, before and after contact lens wear and classification.

Variables	*p*-Value	IC (µ_pre_–µ_post_)	Results	Classification
SD_DA Ratio	0.002	(01361;0.5184)	pre > post	|post–pre| < 1
SD_Int. Radius	0.1	No normality	No sig.	|post–pre| < 0.7
SD_SP-A1	0.129	(−0.46355; 0.06355)	No sig.	|post–pre| < 0.8
SD_SSI	0.779	(−0.20054; 0.26418)	No sig.	|post–pre| < 0.4
SD_ARTh	0.986	No normality	No sig.	|post–pre| < 0.3
bIOP	0.135	No normality	No sig.	
CCT	0.013	No normality	Pre < post	

DA Ratio = deflection amplitude ratio; Int. Radius = integrated radius; SP-A1 = stiffness parameter-A1; SSI = stress–strain index; ARTh = Ambrósio horizontal relational thickness; bIOP = biomechanically corrected intraocular pressure; CCT = central corneal thickness; SD = standard deviation.

## Data Availability

The data presented in this study are available on request from the corresponding author. The data are not publicly available due to their containing information that could compromise the privacy of research participants.
